# A 67-Year-Old Male Patient With COVID-19 With Worsening Respiratory Function and Acute Kidney Failure

**DOI:** 10.1016/j.chest.2021.08.045

**Published:** 2022-01-06

**Authors:** Max Melchers, Barbara Festen, Bianca M. den Dekker, Eline R.M. Mooren, Annelien L. van Binsbergen, Sjoerd H.W. van Bree, Moniek Heusinkveld, Roel Schellaars, Jochem B. Buil, Paul E. Verweij, Arthur R.H. van Zanten

**Affiliations:** a Department of Intensive Care Medicine, Hospital Gelderse Vallei, Ede, The Netherlands; b Department of Medical Microbiology, Hospital Gelderse Vallei, Ede, The Netherlands; c Department of Medical Microbiology and Radboudumc-CWZ Center of Expertise for Mycology, Radboud University Medical Center, Nijmegen, The Netherlands; d Division of Human Nutrition and Health, Wageningen University & Research, Wageningen, The Netherlands

## Abstract

A 67-year-old obese man (BMI 38.0) with type 2 diabetes mellitus (DM), chronic atrial fibrillation, and chronic lymphocytic leukemia stage II, stable for 8 years after chemotherapy, and a history of smoking presented to the ED with progressive dyspnea and fever due to SARS-CoV-2 infection. He was admitted to a general ward and treated with dexamethasone (6 mg IV once daily) and oxygen. On day 3 of hospital admission, he became progressively hypoxemic and was admitted to the ICU for invasive mechanical ventilation. Dexamethasone treatment was continued, and a single dose of tocilizumab (800 mg) was administered. On day 9 of ICU admission, voriconazole treatment was initiated after tracheal white plaques at bronchoscopy, suggestive of invasive *Aspergillus* tracheobronchitis, were noticed. However, his medical situation dramatically deteriorated.

## Physical Examination

On day 10 of ICU admission, the patient was still intubated and on pressure support ventilation. His respiratory rate was 45 breaths/min with mild wheezes, rhonchi, and extended expiration at lung auscultation. He had an irregular pulse rate of 160 beats/min and BP of 106/46 mm Hg without vasopressors. After cold shivers, he developed fever (40.9 °C) and became anuric within the following 48 hours.

## Diagnostic Studies

Laboratory results included platelet count of 66 × 10^9^/L (150-400 × 10^9^/L) and total white blood count of 154.4 × 10^9^/L (4.0-11.0 × 10^9^/L) with 86% lymphocytes and 13% neutrophils. Serum creatinine increased from 120 to 265 μmol/L (60-110 μmol/L) within 24 hours, and Pao_2_/Fio_2_ deteriorated from 126 to 78 (reference, >300).

Galactomannan enzyme-linked immunosorbent assay on the BAL fluid was positive, with OD index of 5.8 (Platelia assay, Bio-Rad Laboratories), and cultures revealed *Aspergillus fumigatus*. Histopathologic examination of the biopsy specimen of a white plaque showed signs of aspergillosis without tissue invasion.

Sputum cultured on day 15 of ICU admission showed sporadic growth of *Rhizopus microsporus*.

**Figure 1:**
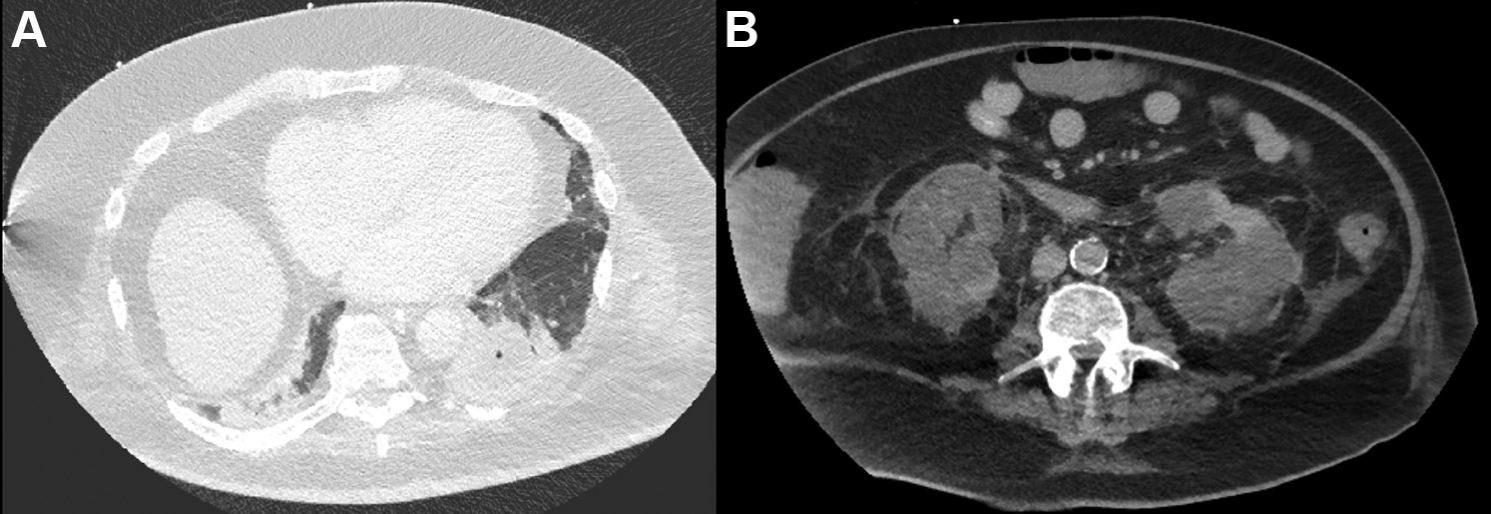
Transversal view of upper body CT scan. A, Pulmonary lesion dorsal in the left lower lobe with signs of pleural effusion and atelectasis; B, Multiple hypodense foci and edema in both kidneys.

A CT scan demonstrated a dense pulmonary lesion in the left lower lobe and hypodense foci in both kidneys, but no rhino-orbital or cerebral abnormalities (Fig 1).

*What is the diagnosis?*

*Diagnosis:* The patient was diagnosed with COVID-19-associated pulmonary aspergillosis (CAPA) and COVID-19-associated disseminated mucormycosis (CAM) with pulmonary and renal involvement

## Discussion

Invasive mucormycosis (IM) is a rare life-threatening opportunistic infection caused by Mucorales molds. The most common species involved are *Rhizopus* species, *Mucor* species, and *Lichtheimia* species. IM manifests in a variety of sites, such as rhino-orbital, rhino-orbital-cerebral, pulmonary, GI, and cutaneous. Renal involvement and disseminated forms are rare. Factors predisposing patients to IM include uncontrolled DM, neutropenia, the use of immunosuppressants, and hematological-oncologic disease. Patients with hematologic-oncologic disease appear to be more susceptible to pulmonary mucormycosis, whereas rhino-orbital-cerebral disease is more frequently seen in patients with uncontrolled DM. Over the last decades, the prevalence of IM has increased because of enhanced application of immunosuppressive therapies and higher incidence of DM worldwide, although systematic surveillance programs are lacking.

Severe COVID-19 infection and its treatment is an emerging risk factor for IM. The risk of developing secondary fungal infections has been attributed to the accumulation of various predisposing factors such as underlying host factors, as defined by the European Organization for Research and Treatment of Cancer and the Mycosis Study Group Education and Research Consortium, including neutropenia, hematological-oncologic disease, solid organ or allogenic stem cell transplantation, and prolonged use of corticosteroids at a therapeutic dose of more than 0.3 mg/kg for at least 3 weeks in the past 60 days. Lytic effects caused by SARS-CoV-2 virus, immune dysregulation, in other words, cytokine storm syndrome and immune paralysis, and concomitant immunosuppressive treatments, glucocorticoids or immunotherapy, have been suggested to play a role in host susceptibility to develop CAPA and also may contribute to the risk of developing CAM. Case series published to date indicate that poorly controlled DM and glucocorticoid treatment are major risk factors for CAM. In addition, immunotherapy, such as the interleukin-6 inhibitor tocilizumab, may contribute to the risk to develop CAM, although its impact on host susceptibility remains unclear. In contrast, the risk for invasive mycosis may be lowered by depressing hyperimmune reactivity, or cytokine storm, whereas conversely immunomodulative therapy may interfere with host response to fungi, thereby increasing host susceptibility for invasive growth.

Most CAM cases are reported in India, which is known to have a high prevalence of poorly regulated DM, but cases in Europe and North and South America have emerged. Rhino-orbital CAM appears as the most frequent manifestation of disease, whereas pulmonary mucormycosis is mainly seen in COVID-19 patients admitted to the ICU. Recently a series of four CAM cases from The Netherlands has been published showing a range of clinical presentations, including patients with and without DM, both in and outside the ICU and with rhino-orbital cerebral, pulmonary, and disseminated disease.

Early recognition of IM is critical for timely initiation of treatment to prevent rapid progression and dissemination. Therefore, clinicians should be aware of CAM in COVID-19 patients developing facial pain, signs of sinusitis, proptosis, or ophthalmoplegia. Subsequently, cranial imaging by CT scan or MRI is essential, followed by endoscopy with biopsy if sinusitis is present. The renal form presents as an acute-onset kidney injury, typically with bilateral involvement, and is mainly seen in disseminated IM. Renal failure occurs because of near-total occlusion of the renal arteries, which can be evaluated by arterial duplex ultrasonography.

**Figure 2:**
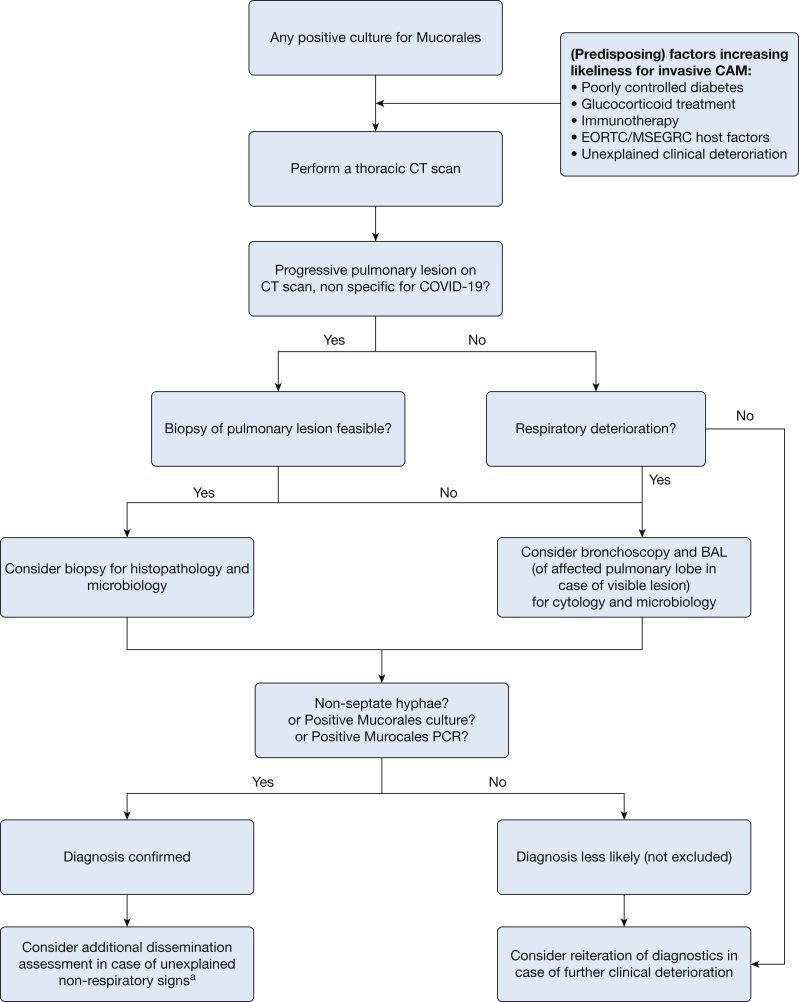
Algorithm for the diagnostic workup for pulmonary COVID-19 associated mucormycosis in the ICU. PCR = polymerase chain reaction. ^a^ie, renal failure, signs of sinusitis, ophthalmoplegia, proptosis, skin ulcers or eschar, or neurological deterioration.

Establishing the diagnosis of pulmonary mucormycosis is challenging, particularly in critically ill COVID-19 patients. Clinical signs of pulmonary mucormycosis are nonspecific, including respiratory deterioration, sputum production, and fever. Furthermore, concurrent CAPA with positive *Aspergillus* cultures may cause misinterpretation of Mucorales colonies in culture and delay escalation to appropriate antifungal therapy. Similar to CAPA, thoracic imaging may show nonspecific lesions and concurrent extensive COVID-19 consolidations. IM also lacks a specific well-validated biomarker, whereas specific molecular tests are not widely available in clinical microbiology laboratories. A recent guideline strongly recommends imaging and biopsy to diagnose IM. This diagnostic strategy may be hampered in COVID-19 patients suspected of pulmonary mucormycosis because of the presence of nonspecific pulmonary lesions and the risk for sampling error. Sampling of the lower respiratory tract through bronchoscopy and BAL may be an alternative, but BAL cultures may remain negative for Mucorales, and tissue invasion is not proven. Positive upper respiratory tract cultures, or sputum or tracheal aspirates, may alert to the potential for IM and prompt a diagnostic workup. In CAPA, positive upper respiratory tract cultures with *Aspergillus* were found not to correspond well with lower respiratory tract cultures (indicating upper respiratory tract colonization), but this correlation remains unclear for Mucorales. Direct microscopy of specimens stained with the fluorescent brightener blankophor P or calcofluor white is recommended. Nonseptate and ribbon-like hyphae with an irregular pattern of branching are suggestive of Mucorales species. Accordingly, any positive culture with Mucorales in COVID-19 patients should be considered clinically relevant and followed by a diagnostic assessment in case of clinical deterioration, particularly when predisposing factors for IM are present (Fig 2).

Current IM guidelines recommend a treatment strategy involving early surgical debridement, regardless of the infection site, and systemic antifungal therapy. Liposomal amphotericin B is the antifungal treatment of choice for all sites of organ involvement. Isavuconazole and posaconazole are considered alternative treatment options and are recommended in case of renal failure. Despite these efforts, IM has a high mortality rate, depending on underlying conditions, infection site, and the ability to perform surgery. In CAM, mortality is reported in half of the patients, with mortality rates ranging between 24.3% and 81% depending on clinical manifestation.

### Clinical Course

After the positive sputum culture with *R microsporus,* voriconazole was switched to isavuconazole, and nebulized amphotericin-B was added. The patient developed mild and coffee-colored hemoptysis, and bronchoscopy was repeated on day 24, delivering negative BAL cultures of the right lower lobe for fungi, and isavuconazole was switched back to voriconazole. Additionally, culture of an ultrasound-guided biopsy of the pulmonary lesion on day 30 remained negative. Subsequent cultures of sputum, obtained during antifungal treatment, remained negative for fungi.

**Figure 3:**
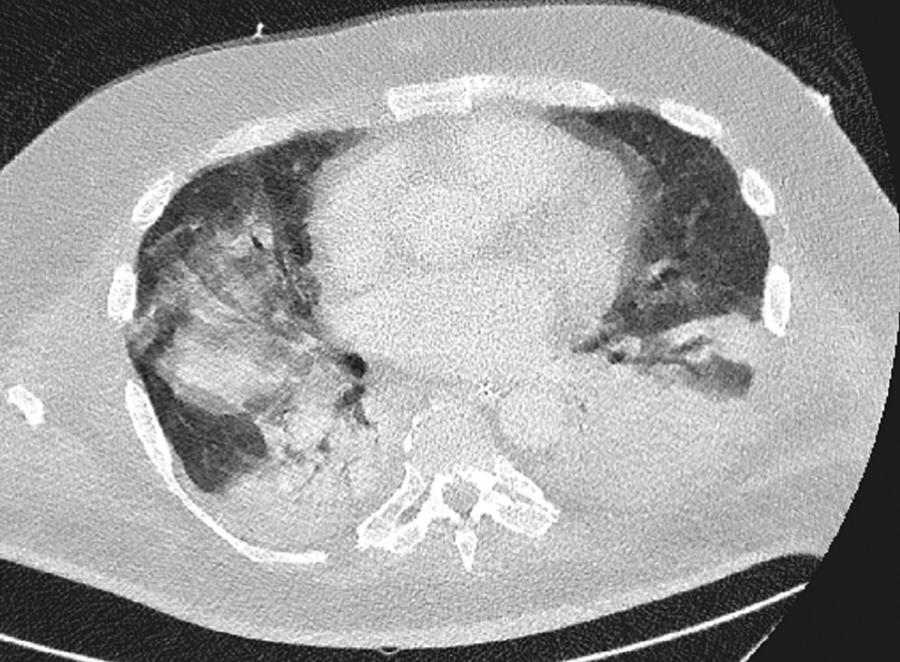
Transversal thoracic CT scan obtained on day 37 of ICU admission, revealing progression of the dense pulmonary lesion dorsal in the left lower lobe.

**Figure 4:**
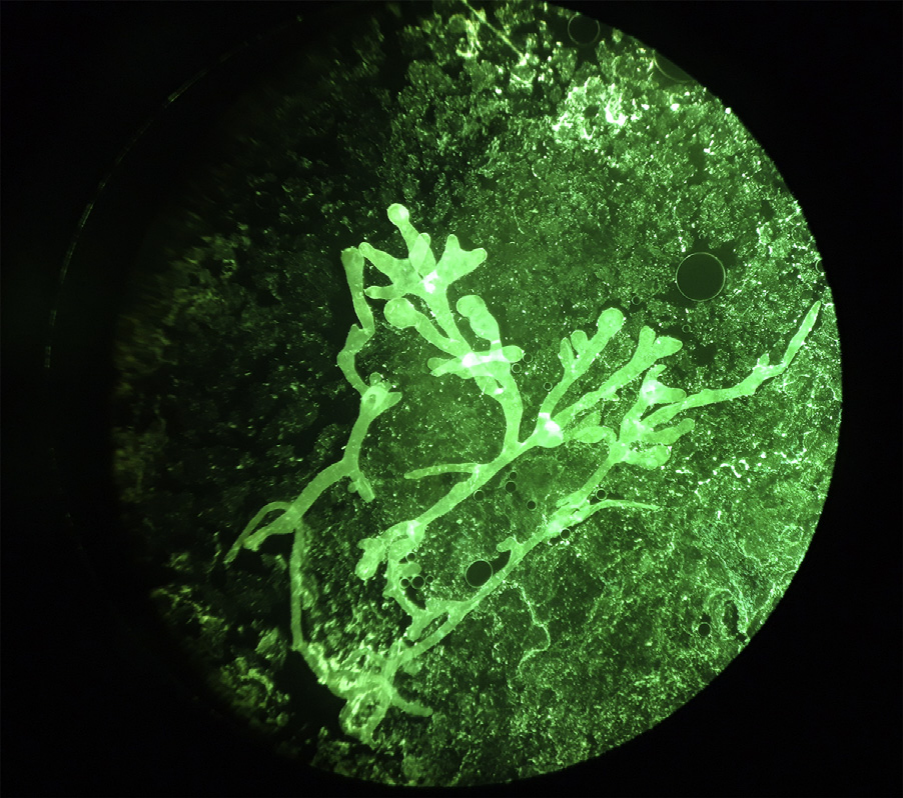
Microscopic image taken with 400× magnitude fluorescent microscope after staining the urine obtained on day 41 with blankophor P, revealing branching, nonseptate hyphae.

**Figure 5:**
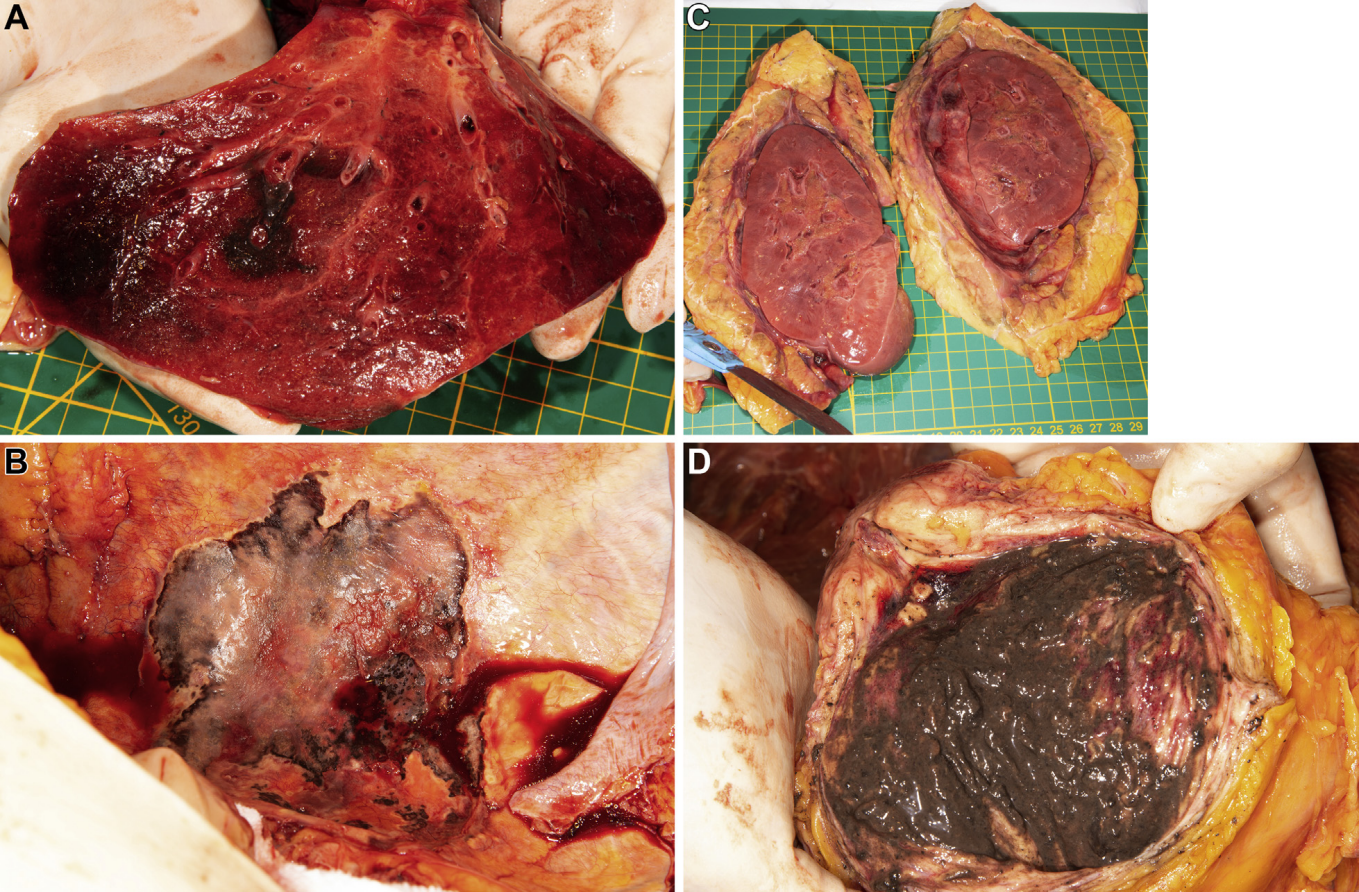
Pictures retrieved at autopsy. A, left lower lobe showing diffuse consolidation with perivascular and bronchial hemorrhagic lesions; B, left lower pleura parietal showing a massive black and grey lesion; C, left and right kidney, both enlarged with several scattered white-yellow plaques and perirenal necrosis and inflammation; D, View on the inside of the bladder covered with black substance.

Reiteration of the thoracic CT scan showed progression of the pulmonary lesion (Fig 3), and voriconazole was switched back to isavuconazole. Later, liposomal amphotericin B was added to the antifungal regimen. The patient was considered ineligible for thoracic surgery because of his poor medical condition. He developed progressive liver and respiratory dysfunction, cardiac arrhythmias, and thrombocytopenia. Furthermore, bladder washout returned black-colored urine. Direct microscopy using blankophor P showed clusters of branching, nonseptate hyphae consistent with Mucorales (Fig 4). Eventually, all treatment was discontinued on day 43 of ICU admission because of progressive treatment failure, and the patient died. Autopsy revealed a necrotizing lesion in the left lower pulmonary lobe (Fig 5A) with hyphal infiltration into the left parietal pleura (Fig 5B), and necrotizing inflammation of the kidneys (Fig 5C), with extension into the abdominal aorta and cystitis nigra (Fig 5D). Direct staining of postmortem bladder-, kidney- and left lung biopsies revealed massive fungal hyphae, and all grew *R microsporus*, confirming the diagnosis of disseminated CAM.

## Clinical Pearls

1. *IM is a rare but potentially lethal co-infection in patients with COVID-19 that appears to be emerging.*
2. *Careful selection of immunosuppressive therapy to treat critically ill COVID-19 patients prone to opportunistic infections is warranted.*
3. *Establishing the antemortem diagnosis of pulmonary CAM is challenging because of the lack of a validated biomarker, false-negative BAL cultures, concurrent CAPA, and nonspecific thoracic CT lesions.*
4. *Positive Mucorales cultures from the respiratory tract in COVID-19 patients should alert the clinician for possible pulmonary CAM. In case of unexplained clinical deterioration and presence of predisposing factors for IM, aggressive therapy following prompt diagnostic workup, should be considered.*
